# The initial report of prospective study for maternal and fetal outcomes to investigate the safety of multifetal pregnancy reduction in Japan

**DOI:** 10.1111/jog.70072

**Published:** 2025-10-01

**Authors:** Nao Wakui, Masayuki Endo, Ayaka Shima, Aiko Okada, Tatsuya Miyake, Shoko Sugao, Kazuya Mimura, Tadashi Kimura, Michiko Kodama

**Affiliations:** ^1^ Obstetrics and Gynecology, Graduate School of Medicine The University of Osaka Suita Japan; ^2^ Children's and Women's Health, Division of Health Science, Graduate School of Medicine The University of Osaka Suita Osaka Japan; ^3^ Clinical Psychology, Graduate School of Human Sciences The University of Osaka Suita Osaka Japan; ^4^ Sakai City Medical Center Sakai Osaka Japan

**Keywords:** multiple pregnancy, patient safety, pregnancy reduction, prospective study, psychological safety

## Abstract

**Aim:**

This study aimed to establish the safety of multifetal pregnancy reduction (MFPR) and examine its complications, patient background, and psychological impact.

**Methods:**

This single‐center, single‐arm, prospective intervention study included 10 women who were treated at our institution between April and December 2024. The indications were triplet or higher‐order multiple pregnancies, as well as twin pregnancies with serious maternal complications. Procedures were performed between 11 and 13 gestational weeks, and the patients were observed for up to 1 week postoperatively. The primary outcome was the completion ratio at hospital discharge, whereas the secondary outcomes were the survival rate of non‐targeted fetuses, the number of adverse events, and the psychological evaluation of the patients at 1 week postoperatively.

**Results:**

Among the 10 patients, one carried twins, seven carried triplets, and two carried quadruplets; half had received ovulation‐induction treatment. The procedure completion rate at discharge was 100%, although one patient required reoperation. The survival rate of non‐targeted fetuses was 89.5% (17/19), and adverse events included vaginal bleeding and complete miscarriage. Maternal state‐anxiety scores decreased significantly postprocedure, whereas depression scores remained elevated both before and after the procedure.

**Conclusions:**

The completion rate of MFPR using echo‐guided transabdominal potassium chloride infusion was high, ensuring short‐term maternal safety. Although no serious adverse event other than one case of total miscarriage was observed, a long‐term safety evaluation is warranted. Moreover, the emotional burden on patients is high before and after the procedure, necessitating the importance of long‐term support.

## INTRODUCTION

With the growing popularity of infertility treatments, the incidence of multiple pregnancies has increased, presenting remarkable challenges in high‐risk obstetrics. For example, the rate of postpartum hemorrhage (singleton: 2%–3% vs. triplet: 9%), preeclampsia (singleton: 2% vs. triplet: 26%),^1^ and preterm birth are increased, and the odds ratio for the occurrence of these complications rises with increasing fetal number. The average number of weeks of gestation is 35.0 weeks for twins, 31.7 weeks for triplets, and 30.3 weeks for quadruplets,^2^ and the incidence of cerebral palsy has increased to 0.65% in twins, 1.71% in triplets, and 5.07% in quadruplts.^3^ In Japan, multiple births have also been identified as a possible risk factor for abusive deaths, with a 1.3‐fold increase per multiple births and a 2.5‐fold increase per family with multiple children.^4^

In 2008, the Japan Society of Obstetrics and Gynecology recommended single‐embryo transfer in assisted reproductive technology (ART), considerably reducing ART‐related multiple pregnancies. Nonetheless, in 2022, multiple birth rates in Japan are 11 045 twins, 157 triplets, and 1.3 quadruplets per one million deliveries.^5^

To mitigate the risks of high‐order multiples, such as large fetal counts, estimated weight discordance, or lethal anomalies, multifetal pregnancy reduction (MFPR) is a possible option. Potassium chloride (KCl) is administered into the fetal cardiac cavity,^6^ or umbilical cord occlusion is achieved via Nd:YAG laser coagulation or radiofrequency ablation.^7^ Although the probability of miscarriage of all fetuses due to MFPR is approximately 5%,^8^ the procedure is performed considering the balance between its risks and the adverse effects of multiple pregnancies on the mother.

In 2003, the Reproductive Medicine Subcommittee of Japan's Health Sciences Council discussed the issue, stating that “the response to multiple pregnancies through reproductive medicine should prioritize the prevention of multiple pregnancies, and fetal reduction should not be performed in principle.” However, they also acknowledged that “completely preventing the occurrence of multiple pregnancies is challenging, and we do not oppose fetal reduction itself,” although no legislation or specific implementation system has been established.^9^ Since then, there has been no public debate regarding this procedure. In Japan, MFPR remains a “hidden” issue, and no one has openly challenged the ambiguities within the abortion law (now known as the Maternal Health Act), which was originally established in 1948.

Although MFPR has been performed at private clinics, few facilities have verified or evaluated its prognosis. In addition, patients and their families who need MFPR are forced to make decisions within a limited timeframe, experiencing guilt and social isolation while lacking access to the necessary information and appropriate psychological support.

To confirm the feasibility and safety of the procedure, as well as to examine its psychological impact in Japan, we conducted a prospective study on maternal and fetal outcomes to investigate the safety of MFPR (PROMISe‐MFPR).

## METHODS

### Study design

This single‐center, single‐arm, prospective intervention study was conducted with the approval of the Institutional Review Board for Clinical Research, Osaka University Graduate School of Medicine (document number: 852), and the Ethical Review Board of Osaka University Hospital (23103‐4). As this study was positioned as an early safety trial and the primary objective was to evaluate surgical completion rates, no statistical significance was assigned to the number of patients included in the study. Considering the expected frequency of cases and study duration, we targeted 10 patients (Table S1).

The inclusion and exclusion criteria of patients are as follows:

Inclusion criteria (those who met all of the following requirements were considered “eligible”)

- Pregnant women who visited our department before 14 gestational weeks.
- Able to provide written consent to participate in the clinical research with her partner.
- Aged between 18 and 50, with a partner ≥18 years.
- Multiple pregnancies of triplets or more, or severe complications that would make the continuation of a twin pregnancy extremely harmful to the health of the mother.
- No vaginal bleeding.
- >20 mm of cervix length.
- No rupture of membranes.

Exclusion criteria (those who met one of the following requirements were considered “ineligible”)

- Positive for HIV antibody, HCV antibody, or HBs antigen.
- With psychiatric symptoms that make participation and continuation in the study difficult.
- Poor general condition: class 3, 4, 5, or 6 in the American Society of Anesthesiology Physical Status Classification.^10^

The primary outcome was the completion ratio of MFPR at discharge from the hospital, and secondary outcomes were as follows: (1) the ratio of non‐targeted fetuses alive 1 week postoperatively; (2) the total number of adverse events during the first postoperative week, including vaginal bleeding, rupture of membranes (ROM), threatened miscarriage, intrauterine infection, maternal death, fetal death (including the death of all children not undergoing abortion), and other adverse events; and (3) psychological evaluation of the patients 1 week postoperatively.

### Treatment protocols and outcomes

#### Feasibility assessment for MFPR

After confirming the pregnancy status of the patients at the time of the outpatient visit to our department, we consulted the clinical ethics board of the hospital, which was made up of experts from other disciplines. Based on the opinion of the board, the MFPR was explained to the patient and her family several times. After written consent was obtained, the patients underwent a preoperative examination to confirm their suitability for the study.

The preoperative assessment items included the following: patient background information (age, body mass index [BMI] at non‐pregnancy, expected date of delivery, number of parities, comorbidities, and results of tests for syphilis, hepatitis, and HIV infection); method of conception (presence of infertility treatment, method of infertility treatment, type and amount of drugs used, and number of embryos transferred if in vitro fertilization); and ultrasound evaluations (number of fetuses and diagnosis of chorionicity and amnioticity). In addition to the above, the following evaluations were performed within 1 week of the date of MFPR, and the results were collected: maternal psychological evaluation (Japanese version of State–Trait Anxiety Inventory‐Form JYZ [STAI‐JYZ], Japanese version of the Center for Epidemiologic Studies Depression Scale [CES‐D]), general condition (BMI, blood pressure, degree of edema, general condition: ASA classification), vaginal examination findings (cervical length, presence of ROM, and presence of vaginal bleeding), urinalysis (urine protein and sugar), blood counts (white blood cell count, neutrophil fraction ratio, red blood cell count, hemoglobin concentration, hematocrit level, and platelet count), ultrasound findings (number of viable fetuses and presence of subchorionic hematoma [SCH]), presence of findings suspicious for abnormal pregnancy (intrauterine infection or signs of threatened miscarriage), and other abnormal self and laboratory findings. The psychological evaluations were conducted by clinical psychologists.

#### Evaluation process before and after MFPR during hospitalization

Patients were admitted the day before surgery, and ultrasound examinations were performed to confirm the number of viable fetuses and membranous diagnoses. We reconfirmed whether the patients and their partners wished to undergo surgery.

**FIGURE 1:**
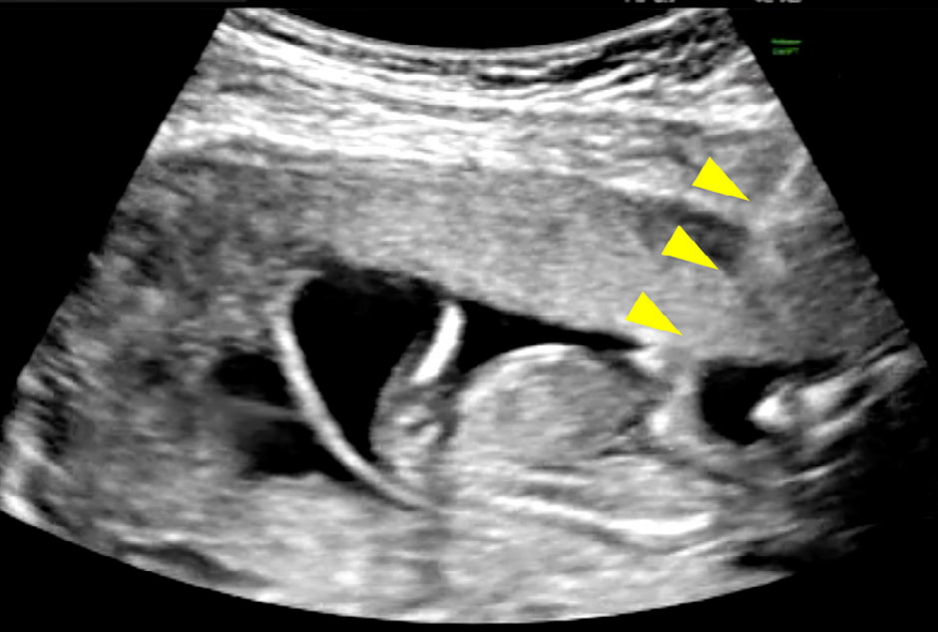
Transabdominal ultrasound‐guided fetal cardiac cavity puncture (arrows indicate puncture needle).

Surgery was performed in the operating room primarily under intravenous anesthesia with propofol. An obstetrician managed the anesthesia, monitoring maternal blood pressure, heart rate, oxygen saturation, and level of consciousness, and administered additional doses as needed. Two obstetricians, including at least one physician designated under the Maternal Health Act, entered the sterile field; one operated the ultrasound machine, and the other performed the puncture. Both physicians were experienced in intrauterine fetal interventions, such as thoracic–amniotic shunt placement and fetal blood transfusion. A 23‐gauge needle was inserted under transabdominal ultrasound guidance into the fetal cardiac cavity (Figure 1) or, if access was difficult, into the thoracic cavity, and a 15% KCl solution (1 mEq/mL) was then injected. After confirming cardiac arrest of the targeted fetus, the needle was withdrawn and viability of non‐targeted fetuses was assessed. We documented the number of surviving fetuses at surgery onset, anesthetic type and dose, duration of anesthesia, operative time, and MFPR completion.

The following morning, examination confirmed cardiac arrest in the targeted fetuses, the continued viability of the non‐targeted fetuses, and absence of maternal adverse events—vaginal bleeding, SCH, intrauterine infection, ROM, threatened miscarriage, maternal death, or other self‐ or other abnormal symptoms. Patients who completed MFPR were discharged later that same day.

#### Postoperative evaluations after discharge from the hospital

One week after surgery (visits were allowed within 4 days before and after surgery), the patients were seen in an outpatient clinic for an internal examination, ultrasound, and psychological examination (STAI‐JYZ and CES‐D). The evaluated outcomes were cervical length, presence of adverse maternal events, and survival of non‐targeted fetuses.

#### Our team's approach to the timing of MFPR and selection of the targeted fetus

The timing for MFPR was set at 11–13 gestational weeks because early miscarriages or the possibility of natural selection for multiple pregnancies can be expected up to approximately 8–10 weeks.^11^

After MFPR, pregnancies were uniformly reduced to twins except in cases of serious maternal complications (cardiac, renal, or autoimmune). Targeted fetuses were then selected based on medical considerations: feasibility of safe transabdominal puncture, prioritizing fetuses on the ventral and basolateral uterine surfaces to avoid injury to the amniotic sac and chorionic villi of adjacent fetuses, and avoidance of fetuses sharing the same chorionic villus to prevent KCl diffusion into non‐targeted fetuses. We did not base fetal selection on genetic or sex determination. However, when maternal health could not tolerate multiple pregnancies, fetuses with morphological malformations (anencephaly or fetal hydrops) identified on preoperative ultrasound were chosen for reduction.

### Evaluation methods for psychological tests (STAI‐JYZ and CES‐D)

#### STAI‐JYZ

The Japanese version of the STAIJYZ^12^ was used to assess anxiety. In Japan, scores at or above the 70th percentile are classified as “high anxiety,” with cutoff values for women set at state‐anxiety ≥55 and trait‐anxiety ≥50. These thresholds are based on a standard female college student population; because few Japanese studies have administered the STAIJYZ to pregnant women, we applied these cutoff values in the present study.

#### CES‐D

The Japanese version of the CES‐D^13^ was used to assess depressive symptoms. A cutoff of 16 points is commonly used to screen for clinical depression risk (sensitivity 63.6%–100%, specificity 53%–100%).^14^

### Statistical analysis

Data are presented as medians (maximum–minimum). To compare measurements between the two groups, the Wilcoxon signed‐rank test was used for non‐parametric analysis. Fisher's exact test (bilateral verification) was used to analyze the number of patients who exceeded the cutoff value on the psychological tests. Statistical significance was set at *p* < 0.05. All statistical analyses were performed using the JMP Pro 17 software (SAS Institute, Cary, NC).

## RESULTS

### Patient characteristics and general conditions

All patients were diagnosed with multiple pregnancies at other institutions and referred to our hospital for consultation regarding MFPR after receiving information from their attending doctors or reading our website, where we were conducting clinical research on MFPR. Patient characteristics and general conditions are summarized in Table 1. General infertility treatment (not ART) with follicle stimulation and ovulation induction was performed in half of the cases. The median age was 30.5 years, and seven patients were primiparas. Embryo transfers were performed in three cases, two of which had two embryos transferred and one of which had three embryos transferred, resulting in triplets. In one case, MFPR was performed for twin pregnancies because of maternal complications. The general preoperative condition, hematological evaluation, and urinalysis results of the patients were normal (data not shown).

**TABLE 1:** Patient characteristics and general conditions.

Case no.	Age	BMI at non‐pregnancy	Parity	Methods of conception	In details of infertility treatment	Medical history/comorbidities	Diagnosis of multiple pregnancy^a^
1	32	22.8	1	SP	–	Hypertrophic cardiomyopathy	DD twins
2	26	30.9	0	TI	Ovulation induction was performed using clomiphene hydrochloride and rFSH preparations, with three follicles measuring 18 mm or more.	–	QQ quadruplets
3	28	16.8	0	SP	–	–	DT triplets
4	40	23.4	0	ET	After two SET failed to result in pregnancy, DET was performed this time.	–	DT triplets
5	30	19.5	0	IUI	Six TIs and one IUI were performed but did not result in pregnancy. Ovulation induction was performed with one follicle over 20 mm in each bilateral ovary and multiple other follicles developing.	–	QQ quadruplets
6	38	24.1	0	ET	Three SETs, one 2 step ET, and one DET failed to result in pregnancy. This time, 2 step ET (one early embryo and one blastocyst) was performed.	–	DT triplets
7	37	19	1	ET	After two SET failed to result in pregnancy, 2 step ET (one early embryo and two blastocyst) was performed.	Hernia of intervertebral disk	TT triplets
8	28	19.2	0	IUI	Three large follicles had developed before ovulation stimulation, and IUI was performed with the patient's consent after explaining the risk of multiple pregnancy.	–	TT triplets
9	25	25.4	0	TI	Ovulation induction was performed with one 22 mm follicle and several smaller follicles.	PCOS OHSS	TT triplets (vanishing quadruplets)
10	31	20.1	1	TI	Details of the situation of ovulation induction are unknown.	–	TT triplets
Median (min–max)	30.5 (25–40)	21.5 (16.8–30.9)					

Abbreviations: DET, double embryo transfer; ET, embryo transfer; IUI, intrauterine insemination; OHSS, ovarian hyperstimulation syndrome; PCOS, polycystic ovarian syndrome; SET, single embryo transfer; SP, spontaneous pregnancy; TI, timed intercourse.

^a^ Number of chorionic villus/ number of amnions/type of multiple pregnancy.

### Details of MFPRs and pre‐ and postoperative obstetrical findings

None of the fetuses exhibited anencephaly or fetal hydrops. The details of the surgery are presented in Table 2. Ketamine hydrochloride was administered in case 1 (hypertrophic cardiomyopathy) to avoid excessive hypotension. Additional propofol was administered because of inadequate sedation. Most cases involved three fetuses; however, two cases involved four fetuses. Each targeted fetus was administered 1.0–2.0 mEq of KCl. However, in case 7, the needle could not easily physically advance into the heart cavity of the targeted fetus. Consequently, 3.0 mEq of KCl solution was injected into the thoracic cavity; however, cardiac arrest was not achieved. The puncture angle was adjusted again, and an additional 1.0 mEq was administered into the cardiac cavity, resulting in cardiac arrest of the targeted fetus. Negative pressure was applied once the puncture needle had reached the fetal cardiac cavity; however, backflow of blood was confirmed in approximately half of the fetuses. In all cases, MFPRs were completed after confirming the cardiac arrest of the targeted fetuses. In case 1, the targeted heartbeat of the fetus resumed the next day, and reoperation was performed after consultation with the patient and her partner; therefore, the two operations are described as 1‐1 and 1‐2. In case 1‐1, the operation was terminated after confirming cardiac arrest of the targeted fetus for 1 min, whereas in case 1‐2, it was confirmed for 2 consecutive min. All procedures in case 1 were performed using the same protocol. The completion ratio of the MFPR at discharge (the primary outcome) was 100%.

**TABLE 2:** Details of multifetal pregnancy reductions (MFPRs) and pre‐ and postoperative obstetric findings.

Case no.	Gestational weeks/days	Type and amount of anesthetic used	Anesthesia/operative time (min)	Type of multiple pregnancy		Amount of KCl used per fetus (mEq)	Reverse bleeding	Completion the day after MFPR/at discharge	Cervical length of before/1 week after MFPR (mm)	1 day/1 week after MFPR				Comprehensive evaluation (any adverse events during observation periods)
				Before/after MFPR	Number of targeted fetuses					Vaginal bleeding	ROM	SCH	Survival of non‐target fetuses	
1‐1	13 + 1	Diazepam 10 mg Ketamine Hydrochloride 40 mg 1% Propofol 50 mg	48/16	DD twins/singleton	1	1	Yes	No/–	60.4/46.2	−/−	−/−	−/−	+/+	No
1‐2	13 + 2	1% Propofol 130 mg	33/22			1	No	Yes/Yes						
2	12 + 1	Ketamine Hydrochloride 40 mg 1% Propofol 300 mg	56/29	QQ quadruplets/DD twins	2	2.0/2.0	Yes/Yes	Yes/Yes	67.4/45.0	−/−	−/−	−/−	+/+	No
3	13 + 0	1% Propofol 260 mg	34/8	DT triplets/MD twins	1	2	Yes	Yes/Yes	68.9/33.0	−/−	−/−	−/−	+/+	No
4	11 + 4	1% Propofol 200 mg	33/9	DT triplets/MD twins	1	2	Yes	Yes/Yes	43.5/52.7	−/−	−/−	−/−	+/−	**Yes (IUFD of all non‐targeted fetuses)**
5	11 + 1	1% Propofol 350 mg	64/30	QQ quadruplets/DD twins	2	2.0/2.0	Yes/No	Yes/Yes	42.5/53.5	−/−	−/−	−/−	+/+	No
6	13 + 3	1% Propofol 520 mg	61/41	DT triplets/MD twins	1	1	No	Yes/Yes	39.1/49.4	−/−	−/−	−/−	+/+	No
7	13 + 0	1% Propofol 310 mg	96/83	TT triplets/DD twins	1	4	Yes	Yes/Yes	46.0/48.1	−/−	−/−	−/−	+/+	No
8	12 + 5	1% Propofol 30 mg	31/16	TT triplets/DD twins	1	1.5	No	Yes/Yes	57.3/60.3	−/−	−/−	−/−	+/+	No
9	12 + 4	Diazepam 5 mg 1% Propofol 240 mg	28/10	TT triplets/DD twins	1	1	No	Yes/Yes	29.2/36.4	−/−	−/−	−/−	+/+	No
10	12 + 2	1% Propofol 180 mg	24/11	TT triplets/DD twins	1	1	No	Yes/Yes	53.3/59.9	−/+ (a little, brown)	−/−	−/−	+/+	**Yes (vaginal bleeding)**
Median or ratio			34/16			2	53.8 (%)	90.9/**100** (%)	49.65/48.75 (*p* value = 1.0^a^)	0/10 (%)	0/0 (%)	0/0 (%)	100/**89.5** (%)	**20.0 (%)**

Abbreviations: IUFD, Intrauterine fetal death; ROM, rupture of membrane; SCH, subchorionic hematoma.

^a^ Wilcoxon signed rank test. Bold: primary and secondary outcomes.

No significant shortening of the cervical length was observed after MFPRs, and no patient had vaginal bleeding, ROM, SCH, or death of non‐targeted fetuses on the day after surgery. On examination, at 1 week postoperatively, case 10 had a slight brownish discharge, and case 4 had intrauterine fetal death of all non‐targeted fetuses. In case 4, we operated on three dichorionic triamniotic triplets and reduced them to monochorionic biamniotic twins. No obvious discrepancy in amniotic fluid volume or crown‐rump length was observed between the twins.

### Psychological evaluation

#### Assessment of anxiety

The results are summarized in Table 3. The median state‐anxiety score was as high as 50 points preoperatively and significantly improved to 41.5 points postoperatively (*p* = 0.0056). However, the percentage of patients exceeding the cutoff value (55 points) did not differ significantly between preoperative and postoperative periods. On the other hand, the trait‐anxiety scores were not significantly different preoperatively and postoperatively, with only one or two patients exceeding the cutoff values in both groups. This suggests that the observation group did not exhibit high levels of organic anxiety compared to the general population.

**TABLE 3:** Changes in psychological evaluations before and after surgery.

Case no.	State–trait anxiety inventory‐JYZ (STAI‐JYZ)							The Center for Epidemiologic Studies Depression Scale (CES‐D)			
	State‐anxiety score (Y‐1)				Trait‐anxiety score (Y‐2)						
	Before MFPR	After MFPR	*p* value		Before MFPR	After MFPR	*p* value		Before MPR	After MPR	*p* value
1	61	57			52	47			17	21	
2	55	40			38	34			12	16	
3	63	47			56	53			31	21	
4	40	34			39	39			14	8	
5	37	31			37	34			21	14	
6	51	46			49	42			21	16	
7	53	45			43	48			19	16	
8	37	31			36	36			6	8	
9	49	43			35	42			30	20	
10	40	25			26	25			6	3	
Median (min–max)	50 (37–62)	41.5 (25–57)	0.0056^a^		38.5 (26–56)	40.5 (25–53)	0.53^a^		18 (6–31)	16 (3–21)	0.083^a^
Number of patients with scores ≥55	3	1	0.58^b^	Number of patients with scores ≥50	2	1	0.50^b^	Number of patients with scores ≥16	6	6	1.0^b^

^a^ Wilcoxon signed rank test.

^b^ Fisher's exact test (bilateral verification).

#### Assessment of depression

The median preoperative score was 18 points, whereas the median postoperative score was 16 points, indicating slight improvement (Table 3). Upon examining the individual data, 3 of 10 participants exhibited an increase of 2–4 points in their depressive scores postoperatively, whereas seven participants demonstrated a reduction ranging from 3 to 10 points. However, the number of patients exceeding the cutoff value was six both before and after surgery, indicating that the patient population was exposed to high levels of depression throughout the observation period.

## DISCUSSION

MFPR should be performed to “protect the life and health of the mother from the risks associated with the continuation of multiple pregnancies,” which is consistent with the principles of the Maternal Protection Law; therefore, we submit abortion reports to the Osaka Prefectural Government (stating in the remarks column that MFPR was performed). Currently, however, few medical institutions in Japan publicly announce their MFPR practices, and the reality remains unclear.

### Assessment of MFPR implementation

The primary outcome (the completion rate of the operation) was 100%, making it a reliable method for MFPR (Table 2). The success rate of umbilical artery infusion of KCL remains approximately 90%,^15^ whereas intracardiac puncture achieved almost a 100% success rate.^15^, ^16^ However, to prevent reoperation, as in case 1, once cardiac arrest of the targeted fetus is confirmed, a sufficient observation time (2 min in this study) should be provided during the operation, and multiple confirmations should be made after the operation and at discharge.

MFPR with KCl infusion has been performed worldwide, with many studies focusing on the timing of surgery at approximately 11–15 weeks.^8^, ^17^ The timing and method used in this study were considered appropriate because the transabdominal route has a lower miscarriage rate than the transvaginal route.^8^, ^17^ These procedures, such as fetal thoracic–amniotic shunt placement or fetal blood transfusion, were performed accurately by multiple surgeons skilled in intrauterine fetal treatment.

### Assessment of physical safety on MFPR

The only adverse maternal event observed in this study was a small amount of genital bleeding, which was acceptable (Table 2). Based on previous reports, we assumed that IUFD, an adverse event on the fetal side, was the most likely adverse event (approximately 5%^8^) and provided information to the family of the patient before participation in the study. In cases of DT triplets, no consensus was reached as to whether a single fetus or MD twins should be retained. Although there are reports that only a single fetus should be retained from the viewpoint of the perinatal prognosis of non‐targeted infants,^18^ case 4 lost all fetuses despite preserving the MD twins for unknown reasons. In principle, the number of fetuses undergoing invasive procedures should be minimized, and decisions should be made carefully based on the general condition of the patient.

One concern in this study was the high maternal anesthetic dose. In all cases, blood pressure never fell below 70/50 mmHg during intermittent intraoperative monitoring; however, several cases required 50–100 mg of propofol before induction of anesthesia, possibly because the mothers were very nervous. Although propofol may transiently suppress neurobehavioral function in neonates at high doses (9 mg/kg) during cesarean section,^19^ no teratogenic effect has been reported, and propofol is considered relatively safe for use in early pregnancy.

### Assessment of psychological safety on MFPR

Several studies have evaluated the psychological status of women and their partners following MFPR. Although most studies found that the frequency of depression was not significantly higher among normal mothers or those who did not undergo MFPR, many cases of feelings of guilt and regret that persisted for several years were observed, indicating the importance of providing long‐term postpartum support to mothers and partners.^20^, ^21^, ^22^ However, no prospective studies have compared psychological changes before and after MFPR, and this is the first of such reports.

The results of the psychological tests are presented in Table 3. Postoperative reductions in state‐anxiety scores were likely attributable to relief following MFPR completion and survival of non‐targeted fetuses. In contrast, because the CES‐D captures symptoms over the “last week,” elevated scores may reflect preoperative anxiety about MFPR, postoperative concern for non‐targeted fetuses, and guilt regarding the targeted fetus until the 1‐week follow‐up. Although many patients initially exceeded the CES‐D cutoff, most exhibited postoperative score declines, suggesting that anxiety and depression may diminish over time. However, a Japanese study of CES‐D scores among 2nd trimester gestational diabetic pregnant women reported a mean score of 9.3 (0–42), with 8.0% of those exceeding the cutoff value (16 points),^23^ suggesting that the degree of depression in pregnant women undergoing MFPR is quite strong.

Participants scoring above cutoffs, or those lacking access to psychological support, received individualized psychoeducation from clinical psychologists. Patients with high anxiety and persistent depression throughout pregnancy may require referral to mental health services. Previous research shows that maternal anxiety can influence physiological and metabolic processes, psychosocial adaptation, and fetal brain development.^24^, ^25^, ^26^ Although anxiety is a natural response during pregnancy, clinicians should remain mindful of its potential long‐term effects.

The long‐term psychological burden may be exacerbated by an inability to express grief for the targeted fetus. Emotional and psychological support beyond the gestational period is therefore critical,^27^ and follow‐up studies are warranted.

Although the planned enrollment period was 2 years, recruitment concluded in 9 months, suggesting greater than expected demand for MFPR consultation.

### Limitations

The study included only 10 cases, and the observation period was limited to 1 week postoperatively to collect reliable outcomes. However, to further evaluate the safety of the procedure, following the outcomes of mothers and fetuses at least until after delivery is essential; such an observational study is currently being planned.

The emotional burden on mothers before and after MFPR is severe, and support should continue until after delivery. It is important to provide support not only to the mother herself but also to her partner and family. From this perspective, establishing a system to conduct a nationwide multicenter collaborative study in the future is essential.

In conclusion, this is the first prospective study on MFPR in Japan. MFPR was successfully performed by skilled surgeons using echo‐guided transabdominal KCl infusion with careful monitoring. Short‐term maternal and fetal safety was confirmed. However, a longer observation period is needed to assess the long‐term maternal and fetal safety. However, it became clear that even within a short observation period of 1 week postoperatively, the emotional burden on the patient was significant. The importance of long‐term psychological support, including that from partners and family members, has been emphasized.

## AUTHOR CONTRIBUTIONS

**Nao Wakui:** Conceptualization; data curation; investigation; project administration; visualization; writing – original draft. **Masayuki Endo:** Conceptualization; investigation; methodology; funding acquisition; supervision; writing – review and editing. **Ayaka Shima:** Investigation. **Aiko Okada:** Conceptualization; investigation. **Tatsuya Miyake:** Conceptualization; investigation. **Shoko Sugao:** Methodology; investigation; writing – original draft. **Kazuya Mimura:** Conceptualization; investigation; supervision; writing – review and editing. **Tadashi Kimura:** Conceptualization; methodology; writing – review and editing; supervision. **Michiko Kodama:** Supervision; writing – review and editing.

## CONFLICT OF INTEREST STATEMENT

The authors declare no competing financial interests or personal relationships that may have influenced the work reported in this study. Dr. Michiko Kodama is an Editorial Board member of this submitted JOGR Journal and a co‐author of this article. To minimize bias, she was excluded from all editorial decision‐making related to the acceptance of this article for publication.

## Supporting information


**TABLE S1:** The raw data of the study.

## Data Availability

The data that support the findings of this study are available in Table S1.
